# Complication Probability Models for Radiation-Induced Heart Valvular Dysfunction: Do Heart-Lung Interactions Play a Role?

**DOI:** 10.1371/journal.pone.0111753

**Published:** 2014-10-31

**Authors:** Laura Cella, Giuseppe Palma, Joseph O. Deasy, Jung Hun Oh, Raffaele Liuzzi, Vittoria D’Avino, Manuel Conson, Novella Pugliese, Marco Picardi, Marco Salvatore, Roberto Pacelli

**Affiliations:** 1 Institute of Biostructure and Bioimaging, National Council of Research (CNR), Naples, Italy; 2 Department of Advanced Biomedical Sciences, Federico II University School of Medicine, Naples, Italy; 3 Department of Medical Physics, Memorial Sloan Kettering Cancer Center, New York, New York, United States of America; 4 Department of Clinical Medicine and Surgery, Federico II University School of Medicine, Naples, Italy; ENEA, Italy

## Abstract

**Purpose:**

The purpose of this study is to compare different normal tissue complication probability (NTCP) models for predicting heart valve dysfunction (RVD) following thoracic irradiation.

**Methods:**

All patients from our institutional Hodgkin lymphoma survivors database with analyzable datasets were included (n = 90). All patients were treated with three-dimensional conformal radiotherapy with a median total dose of 32 Gy. The cardiac toxicity profile was available for each patient. Heart and lung dose-volume histograms (DVHs) were extracted and both organs were considered for Lyman-Kutcher-Burman (LKB) and Relative Seriality (RS) NTCP model fitting using maximum likelihood estimation. Bootstrap refitting was used to test the robustness of the model fit. Model performance was estimated using the area under the receiver operating characteristic curve (AUC).

**Results:**

Using only heart-DVHs, parameter estimates were, for the LKB model: *D_50_* = 32.8 Gy, *n* = 0.16 and *m* = 0.67; and for the RS model: *D_50_* = 32.4 Gy, *s* = 0.99 and *γ* = 0.42. AUC values were 0.67 for LKB and 0.66 for RS, respectively. Similar performance was obtained for models using only lung-DVHs (LKB: *D_50_* = 33.2 Gy, *n* = 0.01, *m* = 0.19, AUC = 0.68; RS: *D_50_* = 24.4 Gy, *s* = 0.99, *γ* = 2.12, AUC = 0.66). Bootstrap result showed that the parameter fits for lung-LKB were extremely robust. A combined heart-lung LKB model was also tested and showed a minor improvement (AUC = 0.70). However, the best performance was obtained using the previously determined multivariate regression model including maximum heart dose with increasing risk for larger heart and smaller lung volumes (AUC = 0.82).

**Conclusions:**

The risk of radiation induced valvular disease cannot be modeled using NTCP models only based on heart dose-volume distribution. A predictive model with an improved performance can be obtained but requires the inclusion of heart and lung volume terms, indicating that heart-lung interactions are apparently important for this endpoint.

## Introduction

Technological advances in radiation therapy have increased user control over organ-at-risk dose distributions. In a modern radiotherapy setting, radiobiological models potentially play an essential role and normal tissue complication probability (NTCP) modeling may help to identify the optimal plan that minimizes side effects for individual patients.

The toxicity endpoint that have been modeled include radiation-associated cardiac disease [1]. Indeed, late cardiac toxicity is one of the most feared side effects of therapeutic thoracic radiation therapy. Unfortunately, relevant data are limited. Modeling radiation-induced heart disease is hampered both by the relatively low incidence of the complication and the lack of long term results from 3D-based thoracic RT [2]–[4].

Lyman-Kutcher-Burman (LKB) and Relative Seriality (RS) NTCP heart parameter values have been summarized in the QUANTEC Reports dedicated to radiation-dose volume effects on the heart [1]. Those parameters were estimated for endpoints like pericarditis/pericardial effusion, very delayed cardiac mortality, as well as cardiac perfusion defects. Results were extracted from breast cancer and Hodgkin’s lymphoma (HL) patients treated with doses up to 40–50 Gy during the 1970’s and the 1980’s [5]–[7]. Importantly, individual dosimetric data were not always available, and the heart doses were reconstructed as well as possible. Since then, the standard treatment for HL has considerably changed, especially in the last decade [8]–[10].

An additional, well-recognized effect of chest radiation exposure is the development of valvular abnormalities [11], that represent an important endpoint to analyze due to their role in the progressive development from asymptomatic dysfunction to overt heart failure [12]. Dose-based NTCP models such as the LKB and RS [13], [14] models are the most well-known and traditionally accepted methods for predicting toxicity after radiation treatment. However, to date, no LKB or RS NTCP parameters for this specific radiation-induced heart disease are available.

The mentioned traditional NTCP models use only information about the dose distribution and fractionation. However, it has been reported how RT outcomes may also be affected by multiple factors other than the dose [15]. In a previous study [16], using a different modeling philosophy, we have developed a data-driven multivariate logistic predictive model with a good predictive power for the development of radio-induced valvular defects (RVD) in a population of 56 HL survivors. Besides the heart maximum dose and cardiac volume, that study established the statistical importance of lung volume in the risk prediction of heart toxicity supporting the hypothesis of cardiac damage indirectly caused by additional lung volume irradiation [17]–[19].

The aim of the present study is to test the predictive power of traditional LKB and the RS NTCP models for the induction of asymptomatic RVDs using a dataset of HL patients, and to compare this to an updated multivariate logistic regression model fit to the current, larger dataset. We proceed by fitting the NTCP model parameters first from heart dose-volume parameters, and separately lung dose-volume parameters, and then with both heart and lung dose-volume parameters. We also update the multivariate logistic NTCP model, using all available parameters, and compare the results.

## Methods

### Clinical and dosimetric data

The patient dataset reported in this analysis includes all eligible patients from a study of HL survivors [20]. Between 2001 and 2012, 132 total patients entered the clinical study, of whom 90 patients were eligible for the current analysis. Eligibility criteria include availability of complete cardiac toxicity profile before and after RT, lack of any pretreatment cardiac disease, a minimum follow-up of 36 months, and the availability of 3-D treatment dose distributions. The data were analyzed anonymously. Patients and treatment characteristics have been described in detail elsewhere [21], [22], although this cohort now includes 90 patients compared to 56 previously reported on. Briefly, all patients received post-chemotherapy supradiaphragmatic involved-field RT at our radiation oncology department, and were retrospectively reviewed for radio-induced valvular defects (RVD). A diagnosis of RVD was based on the presence of regurgitation and/or stenosis (mild, moderate, or severe) in at least one of the aortic, mitral, tricuspid and pulmonary valves. Patients were followed up for a median time of 80 months (range 38–140 months).

All patients were treated with CT-based 3D conformal RT with a median total dose of 32 Gy (range, 21–41 Gy) in 20 daily fractions of 1.5–1.8 Gy. RT was administered with anterior-posterior/posterior-anterior photon fields (energies: 6 to 20 MV). When needed, the segmented field technique was employed to improve dose uniformity [23]. Multigrid superposition dose calculation algorithm that corrects for the presence of heterogeneous tissues was applied. For all patients, the whole heart was retrospectively contoured on the planning CT-images applying the heart atlas proposed by Feng et al. [24]. Total lung tissue was contoured following RTOG 1106 recommendations [25].

For each patient, dose-volume histogram (DVH) extraction from treatment planning data was performed using the CERR open-source available software platform [26]. In this way, individual DICOM RT plans (doses and contoured heart and lungs) were converted into the Matlab/CERR format for further analysis.

All participants gave written informed consent and the patient data were analyzed anonymously, This retrospective study was approved by the local Ethics Committee (Comitato Etico per le Attività Biomediche, Università Federico II di Napoli, n.222-10).

### Normal tissue complication probability models

The NTCP models used in this study include the Lyman-Kutcher-Burman (LKB) model [14] and the relative seriality (RS) model [13]. LKB and RS modeling was performed taking into account the irradiation of the heart and at a second step the irradiation of the lungs.

### The LKB model

We used the LKB model recast on the concept of generalized equivalent uniform dose (*gEUD*) [27]. This model can be expressed as:
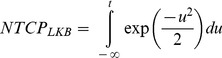





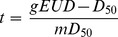
where *D_i_* is the dose and *v_i_* is the relative volume of the *i-*th bin of the differential DVH. The model contains three parameters (*D_50_*, *m, n*)_LKB_. *D_50_* is the uniform dose given to the entire organ volume that results in 50% complication probability, *m* is a measure of the steepness of the slope of the model curve and *n* is a parameter describing the volume dependence of the considered tissue. Small values (<<1) of *n* indicate a sensitivity to the highest dose volume, even if small, whereas values closer to 1 indicate that the response is due to an average of effects across the organ.

### The RS model

In the relative seriality (RS) model, the probability of a complication after irradiation of a relative volume *v_i_* at a dose *D_i_* is given by:
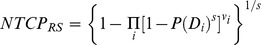


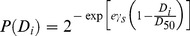
where *P*(*D_i_*) is the probability of complication due to the irradiation of the relative volume *v_i_* at the dose *D_i_* described by an approximation of Poisson statistics. The model contains three parameters (*D_50_, γ, s*)_RS_. *D_50_* has the same meaning as for the LKB model, *γ* is a slope parameter which affects the steepness of the sigmoid shape dose-response curve, and *s* is a parameter that represents the ‘relative seriality’ of organ/tissue under consideration (the ratio of serial subunits to all subunits of the organ). Large values (≈1) of *s* indicate a serial structure and small values (<<1) indicate a parallel structure.

### Correction for fractionation size

The HL patients analyzed in this study were treated with different fraction sizes (1.5 Gy, 1.6 Gy, or 1.8 Gy) other than 2 Gy. In order to compare our results on NTCP parameters estimates with those reported in literature [1] referred to the standard fractionation of 2 Gy, we corrected all heart and lungs DVH bins according to the following equation based on the linear quadratic model [28]:
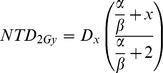
where *NTD_2Gy_* is the normalized total dose to 2 Gy fractions and *D_x_* is the dose for the fractionation scheme *x* Gy. The α/β ratio was set to 3 Gy for the heart [18] and to 4 Gy for the lungs [29].

### Maximum likelihood fitting and confidence intervals

The maximum likelihood (ML) method was employed to find the best fit values for the parameters (*D_50_, m, n*)_LKB_ and *(D_50_, γ, s*)_RS_ of the NTCP_LKB_ and NTCP_RS_, respectively.

The method maximizes the log-likelihood function (LLH):
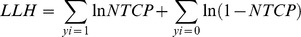
for the known binary outcome (heart valvular toxicity), averaged over the patients (*y_i_*) of the available dataset. Fits were made separately considering heart and lung dose-volume histograms.

The LLH function was numerically maximized by the Nelder-Mead Simplex Method (Matlab implementation: FMINSEARCH function) using an in-house developed library for Matlab. Ninety five percent confidence intervals for parameters estimates were obtained using the profile likelihood method [30]. Following this method, each parameter belonging to the set (*D_50_*, *m, n*)_LKB_ (or equivalently to the set (*D_50_, γ, s*)_RS_) was varied around its ML estimate (optimum LLH) while the other 2 parameters were fixed at their ML estimate. The 95% confidence bounds were determined reducing the maximum LLH by one half of the *χ^2^* inverse cumulative distribution function associated with a 95% confidence level, so as to obtain the iso-likelihood contours in each Cartesian plane of the parameters space (*D50, m, n*), or equivalently, of the (*D_50_, γ, s*) space.

In correspondence to the parameters values belonging to the iso-likelihood contours, a bundle of NTCP curves was calculated and the 95% confidence region for the model fit was thus estimated [31].

Of note, even if a model fits the available dataset, it may fail to be predictive on a different patient population [32]. The bootstrap method was employed to determine the spread in ML estimation of NTCP parameters. The bootstrap resampling method works by refitting the NTCP model using the ML estimation to many pseudo-datasets, which are created by randomly copying or re-copying individual patient datasets from the input data set (20000 bootstrap resamples were used).

### Model evaluation and comparisons

The prediction performance of each NTCP was assessed and the different models were compared. In the comparison, we also included a multivariate logistic NTCP model. In a previous analysis of RVD [16] on a subset (56 patients) of the present HL survivors dataset, we developed a 3-variable logistic regression model consisting of the maximum heart dose (HD_max_), heart volume (HVol), and lungs volume (LVol) given by
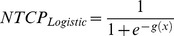





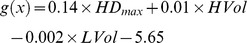
where *HD_max_* was expressed in Gy and *HVol* and *LVol* in cc.

For model evaluation, the comparison between mean predicted rates of RVD by each model and the observed rates for patients grouped according to increasing model risk was performed. Patients were binned according to the NTCP model being considered, with a number of patients in each bin as equal as possible.

Model predictive power was assessed by use of Spearman’s rank correlation coefficient (r_s_). The receiver-operating characteristic (ROC) analysis and the area under the ROC curve (AUC) metrics were employed in order to compare the discriminating ability of each model fit. The discrimination value on the ROC curve, i.e. the cut-off point optimally classifying patients in a binary prediction problem [33], was determined by Youden’s J statistic. The ROC curve was created by plotting the fraction of true positives out of the total actual positives (TPR = true positive rate or sensitivity) vs. the fraction of false positives out of the total actual negatives (FPR = false positive rate or 1-specificity), at various probability threshold settings. Youden's index is the difference between the TPR and the FPR. Maximizing this indicates an optimal cut-off point. ROC curve results were compared using a Z test. Statistical analysis was performed with MedCalc version 12.3.

## Results

**Figure 1:**
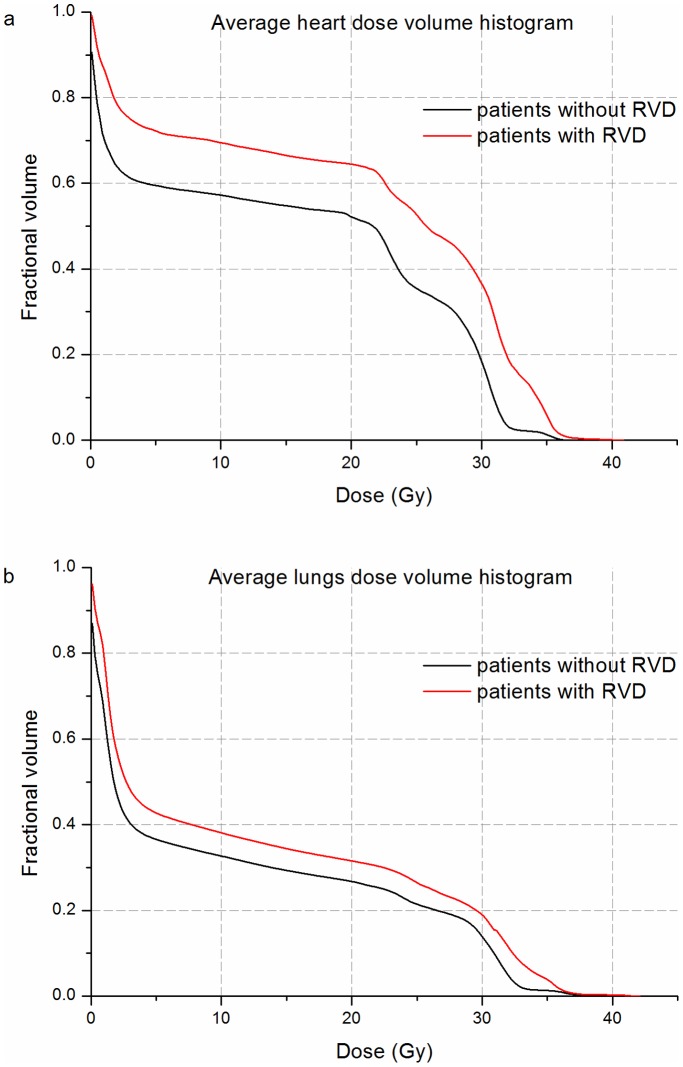
Mean cumulative DVHs for heart (a) and for lung (b). Red line: patients who developed radiation-induced valvular defects; black line: patients who did not develop radiation-induced valvular defects.

Twenty-seven out of 90 patients (30%) experienced at least one kind of RVD. The mean cumulative heart DVHs and the mean cumulative lung DVHs for patients who developed complication and complication-free patients are illustrated in figures 1.a and 1.b. Heart and lung clinic- dosimetric variables are reported in Table S1.

### LKB and RS model fitting based on heart dose-volume parameters

**Figure 2:**
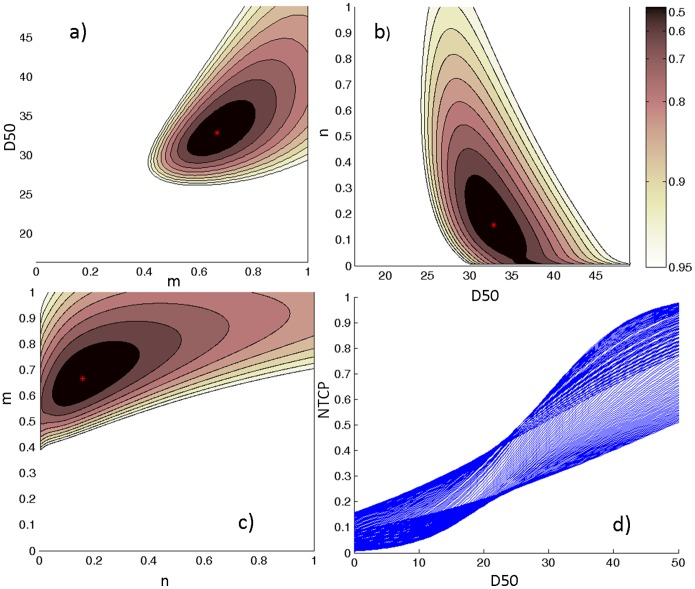
Likelihood estimation values plotted as a function of heart LKB parameters. a) *m* and *D_50_* for a fixed value of *n* = 0.16; b) *D_50_* and *n* for a fixed value of *m* = 0.67; c) *n* and *m* for a fixed value of *D_50_* = 32.8; d) NTCP bundle of curves showing 95% confidence interval region for the model fit. The red point corresponds to the optimum LLH. *Abbreviation*- LKB: Lyman-Kutcher-Burman, *D_50_*: uniform dose given to the entire organ volume that results in 50% complication probability, NTCP: Normal Tissue Complication Probability, LLH: Log-likelihood.

Maximum likelihood estimation and associated confidence intervals (CIs) for the LKB and RS parameters obtained considering the heart irradiation are provided in Table 1. The LKB and RS models showed similar optimal model fits values: the *D_50_* were identical and both volume parameters were consistent with a serial heart architecture (*n* = 0.16 and *s* = 0.99). For the LKB model, the obtained iso-likelihood contours in each Cartesian plane of the parameters space (*D_50_, m, n*) are illustrated in figure 2a–c. The corresponding bundle of NTCP_LKB_ curves are plotted in figure 2d. From Table 1, we can observe a large 95% CI for *D_50_* in both LKB and RS models. The volume parameter 95% CI for the LKB model is quite wide while RS model even includes the whole allowed range for the *s* value, thus suggesting a poor fit of the model to the dataset.

**Table 1:** Parameters estimates and 95% confidence intervals of LKB and RS NTCP models for heart and lung dose volume histograms fitting.

	*D50 (Gy)*	*m*	*n*	*r_s_*	*AUC*	*LLH*
**LKB heart**	32.8	0.66	0.16	0.27	0.67	−51.6
	(25.9, 44.7)	(0.41, 1)	(0.10, 0.89)		(0.56, 0.78)	
**LKB lung**	33.2	0.19	0.01	0.28	0.69	−49.7
	(31.3–35.5)	(0.13–0.32)	(0.01–0.03)		(0.58, 0.78)	
	***D50 (Gy)***	***γ***	***s***	***r_s_***	***AUC***	***LLH***
**RS heart**	32.4	0.42	0.99	0.25	0.66	−52.3
	(22.7, 48.5)	(0.24, 0.62)	(0.0–1.0)		(0.55–0.76)	
**RS lung**	24.4	2.12	0.99	0.26	0.66	−51.1
	(22.3, 26.7)	(0.3–3.8)	(0.67–1.0)		(0.55–0.76)	

*Abbreviation*- LKB: Lyman-Kutcher-Burman, RS: Relative Seriality, NTCP: Normal Tissue Complication Probability, *D_50_*: uniform dose given to the entire organ volume that results in 50% complication probability, r_s_: Spearman’s correlation coefficient, AUC: the area under the receiver operator characteristic curve, LLH: log-likelihood (LLH).

The Spearman’s coefficient and the AUC for each model are also reported in Table 1. The discrimination values were 0.36 and 0.33 for NTCP_LKB_ and NTCP_RS,_ respectively.

Table 2 reports the results for bootstrap cohorts showing that the mean values for heart RS parameters are close to the exact fit to the whole patient cohort.

**Table 2:** Summary of mean and standard deviations of LKB and RS NTCP model parameters obtained with maximum likelihood estimation for bootstrap samples.

	*D50 (Gy)*	*SD*	*m*	*SD*	*n*	*SD*
**LLKB heart**	36.1	5.5	0.67	0.11	0.11	0.12
**LLKB lung**	33.9	1.4	0.22	0.03	0.01	0.02
	***D50 (Gy)***	***SD***	***γ***	***SD***	***s***	***SD***
**RRS heart**	32.7	3.1	0.43	0.07	0.99	0.06
**RRS lung**	24.3	0.83	2.16	0.56	0.99	0.04

*Abbreviation*- LKB: Lyman-Kutcher-Burman, RS: Relative Seriality, NTCP: Normal Tissue Complication Probability, *D_50_*: uniform dose given to the entire organ volume that results in 50% complication probability, SD: standard deviation.

### LKB and RS model fits based on lung dose-volume parameters

**Figure 3:**
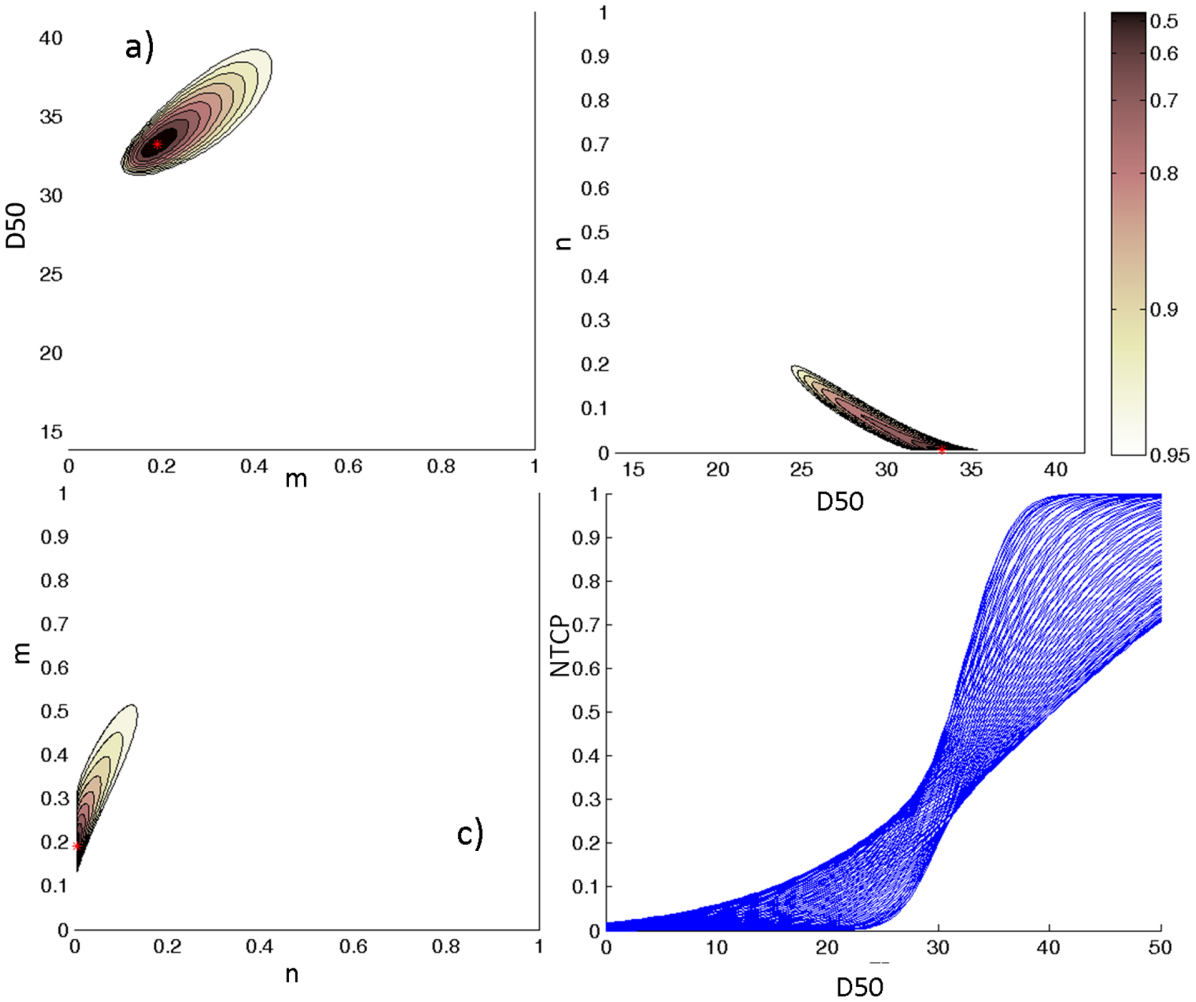
Likelihood estimation values plotted as a function of lung LKB parameters. a) *m* and *D_50_* for a fixed value of *n* = 0.01; b) *D_50_* and *n* for a fixed value of *m* = 0.19; c) *n* and *m* for a fixed value of *D_50_* = 33.2; d) NTCP bundle of curves showing 95% confidence interval for the model fit. The red point corresponds to the optimum LLH. *Abbreviation*- LKB: Lyman-Kutcher-Burman, *D_50_*: uniform dose given to the entire organ volume that results in 50% complication probability, NTCP: Normal Tissue Complication Probability, LLH: Log-likelihood.

Maximum likelihood estimations for the LKB and RS parameters obtained using lungs DVHs are provided in Table 1 along with 95% CI. Iso-likelihood contours and NTCP curve bundle for LKB model are illustrated in figure 3a–d.

The LKB and RS models showed similar volume parameters values suggesting a pronounced (*n* = 0.01 or *s* = 0.99) dependence on the high-dose region when the lung is used to model heart toxicity. Of note, there is a difference of about 10 Gy between the LKB and RS estimates of *D_50_*. The 95% CI values obtained for all three parameters of the lung LKB model showed the very good fit result. For the RS model, only two out of three model parameters had narrow CI, being the *γ* interval of 0.3–3.8.

The r_s_ coefficient and the AUC values for each model are reported in Table 1. The discrimination values were 0.27 and 0.40 for NTCP_LKB_ and NTCP_RS,_ respectively.

Table 2 reports the results for bootstrap cohorts showing the robustness of lung LKB fit procedures.

### LKB combined heart-lung fitting

Beyond parameters estimates for NTCP models for heart valve dysfunction, we explored the possible combined contribution of both heart and lung irradiation to radiation related heart toxicity. In light of the good fitting results obtained for the lung LKB model, we constructed an interaction *gEUD* variable defined as:
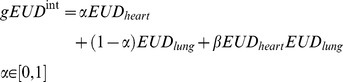
so as to obtain a LKB NTCP*^int^* taking into account the combined organs irradiation. In this way, given the obtained separate estimates of *n_heart_* and *n_lung_* reported in Table 1, the ML method provides the following parameter estimates: *α* = 0.2, *β* = (2.6×10^5^ Gy)^−1^, *D_50_^int^* = 32.6 Gy, and *m^int^* = 0.24. Model prediction performance was only improved slightly, with an AUC of 0.70 (95% CI: 0.59–0.79, discrimination value = 0.34) and an r_s_ = 0.31.

### Model comparisons

The logistic NTCP model previously derived using a subset of patients, when applied on the present extended dataset obtained an r_s_ of 0.50 and an AUC of 0.82 (95% CI: 0.73–0.90, discrimination value = 0.21), thus confirming the good prediction performance of such a model. Of note, the same performance (r_s_ = 0.51 and AUC = 0.82) was obtained refitting the logistic model with the new interaction variable, i.e. *gEUD^int^*, instead of the heart maximum dose originally included in the logistic regression.

**Figure 4:**
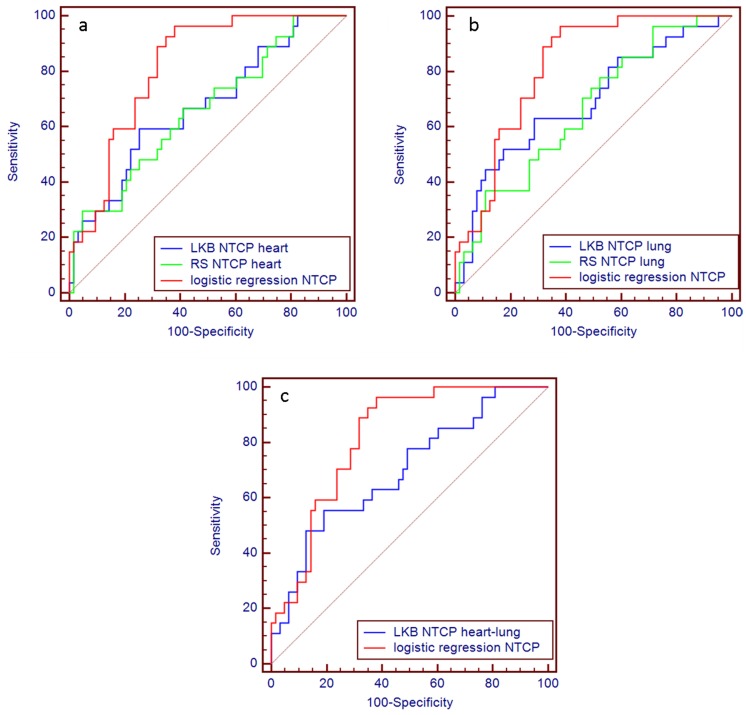
ROC curve comparison. Logistic regression model vs. LKB and RS NTCP model for heart (a) and lungs (b). Logistic regression model vs. combined heart-lung LKB NTCP model (c). *Abbreviation*- ROC: receiver operating characteristic, LKB: Lyman-Kutcher-Burman, RS: Relative Seriality, NTCP: Normal Tissue Complication Probability.

For the standard NTCP models, including also the combined one, AUC values were considerably lower, and varied in an interval between 0.66 and 0.70. Model comparisons are illustrated by ROC curves in figure 4. There is no difference in prediction performance between LKB and RS models (p>0.5). In addition, independently of the organ chosen as the model input, namely heart DVHs or lung DVHs, we obtain similar prediction performances. The data-driven regression logistic NTCP model, however, applied to the present dataset, resulted in being significantly more predictive (p = 0.03) when compared to heart and lung NTCP_LKB_ and NTCP_RS_ models (figure 4a–b). The logistic regression model outperformed also the combined heart-lung LKB model (figure 4c) although the difference between the AUC values approaches the borderline of statistical significance (p = 0.07).

**Figure 5:**
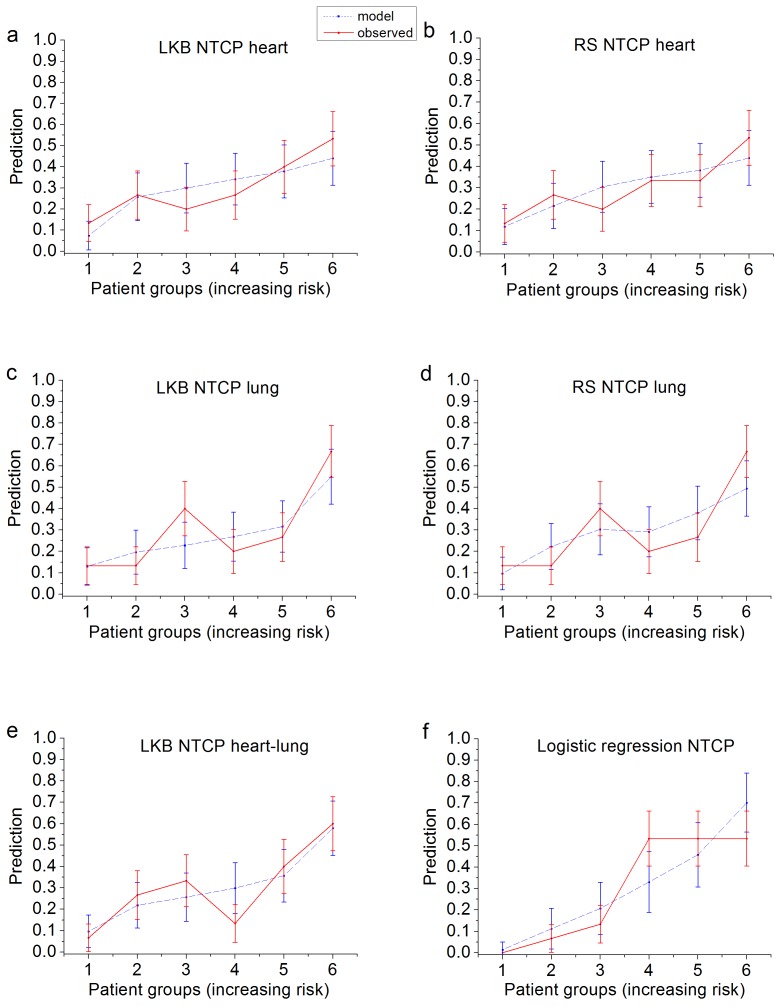
Comparison between the actuarial incidence of radiation-related valvular defects (RVD) in the population and the predicted incidence by each NTCP model. a) heart LKB, b) heart RS, c) lung LKB, d) lung RS, e) three-variable logistic model. Patients were binned according to the considered NTCP model with equal number of patients in each bin. *Abbreviation*- LKB: Lyman-Kutcher-Burman, RS: Relative Seriality, NTCP: Normal Tissue Complication Probability.

The comparison between the predicted incidence of RVD by each NTCP model and the actuarial incidence in the population is shown in figure 5.

## Discussion

The aim of the present study was to explore alternative options for NTCP modelling for radiation-related heart toxicity. We estimated LKB and RS normal tissue complication probability parameters for radiation induced heart valve dysfunction in order to a) provide a comparator to values reported in the literature [1] for radiation induced heart disease different from RVD; b) consider the possible role of lung irradiation in the development of heart disease [19] and c) understand the benefits of a data-driven approach to NTCP modeling of RVD in contrast to phenomenological models such as LKB or RS models.

The clinical importance of radiation-induced heart disease has been well recognized, including the difficulty in the estimation of related risk due to the long latency time. As pointed out by Trott and coworkers [4] cardiovascular radiation damage may occur insidiously as microvascular ischemic radiation injury leading indirectly to focal myocardial damage and myocardial radiation damage is probably secondary to radiation effects in the myocardial microvascular system. The risk of radiation-induced microvascular disease begins to increase 10 years after irradiation and it is progressive with time and a significant increase of risk has been observed with mean heart doses lower than 10% of the generally accepted tolerance dose to the heart [34]. Data for long-term cardiac mortality were derived from retrospective studies of patients treated with outdated techniques [1] and NTCP parameters were based on the relative seriality model giving a *D_50_* of 70 Gy on a Hodgkin’s cohort of patients treated between 1972 and 1985. An estimated value of the *s* parameter equal to one suggested a limited volume dependence. A logistic model [9] has been also applied to dose response in HL in children and adolescents reported in literature [35] estimating a lower *D_50_* of 48 Gy for any cardiac morbidity and a *D_50_* of 40 Gy for valvular disease.

To date, LKB or RS as an alternative modeling for valvular defects has not been performed, although this type of heart defects has been suggested to be possible candidate as early predictor or surrogate for late cardiac morbidities. In the present work, the parameter estimates obtained from the two NTCP models for RVD data fitted as a function of heart dose were mutually consistent, i.e., both of them confirmed a dependence on the highest-dose volumes of the heart. For both models, the *D_50_* value was about 32 Gy. As expected for a mild condition such as valvular disease, we obtained a lower *D_50_* value compared to the reported values for cardiac mortality, while it was well within the 95% CI of the results by reported Maraldo et al. [9].

One of the important aspects to consider in modeling radiation induced normal tissue effects such as RVD is that it represents a complex process involving multiple biological pathways and systems. In particular, radiation-induced fibrosis of the lung and its vessels may affect cardiac functions [36] and a heart-lung interaction in radio-induced toxicity to cardiopulmonary system has been reported [19], [37], [38]. Accordingly, for the first time, a cross modeling exercise was performed: the NTCP models for the radiation induced heart toxicity were also fitted as a function of lung dose. The results of this fitting procedure were comparable or even better (narrower confidence intervals for parameters estimates) than those obtained by heart fitting. For LKB and RS models the *D_50_* values ranged in an interval between 24 and 33 Gy. Of note, we observed a serial behavior of the lung when using heart toxicity as endpoint. This result is different from the generally accepted parallel architecture, with a large volume effect, of the lungs when NTCP models were fit to radiation pneumonitis as endpoint. As a consequence, we can hypothesize a different mechanism of damage and a different contribution of lung irradiation to the heart toxicity potentially due to the difference in patho-physiology, although still unknown.

For all models, the spread in ML estimation was assessed using the bootstrap method (Table 2). This gives a measure of how much the different selection of cases might influence the parameters. Interestingly, the more stable results for all three parameters were obtained again for the LKB model applied to the lungs.

**Figure 6:**
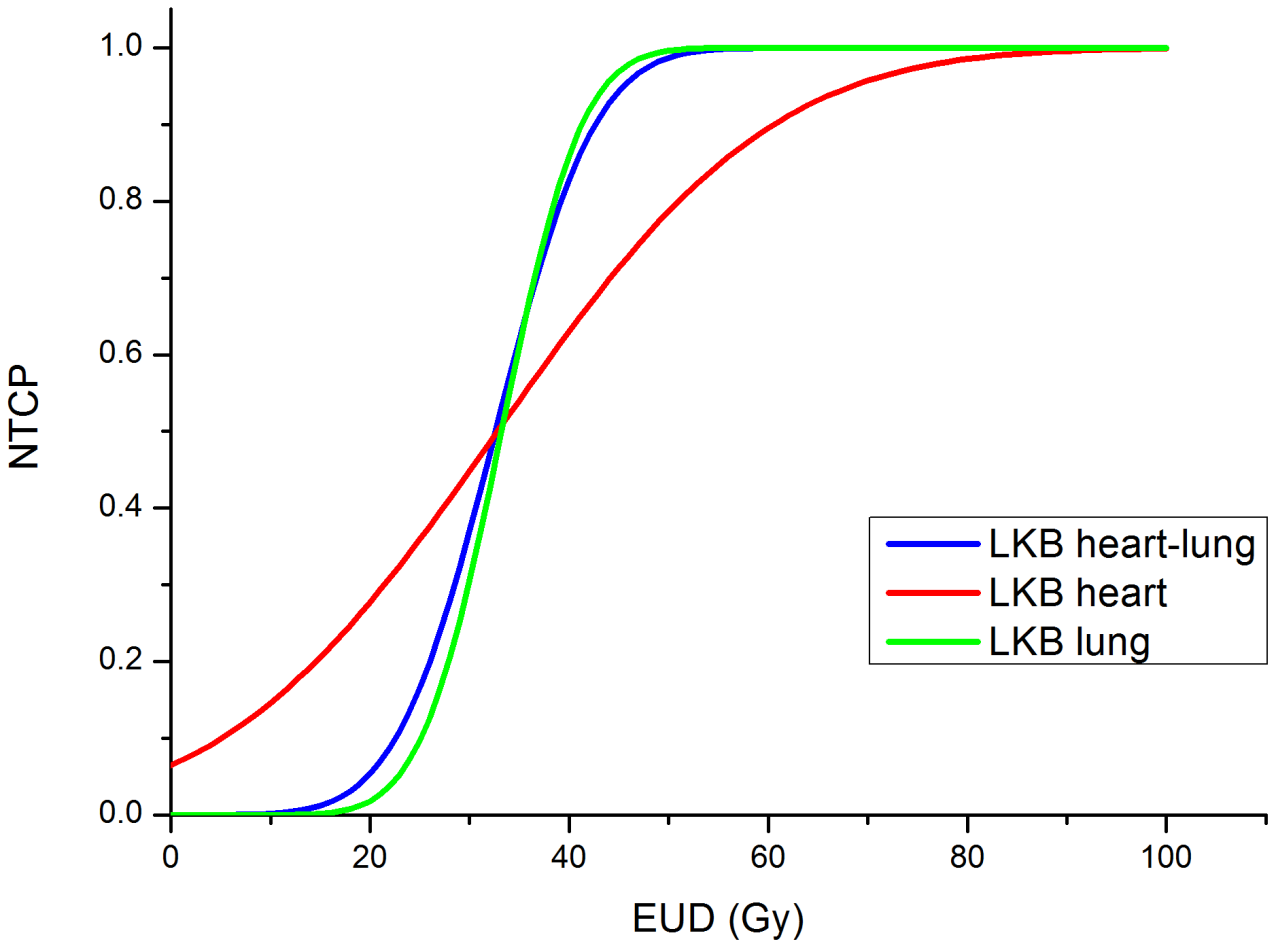
Comparison of different LKB NTCP curves plotted as a function of gEUD. The curve are obtained using parameters estimates by fitting heart (red), lung (green), and combining heart and lung (blue). *Abbreviation*- LKB: Lyman-Kutcher-Burman, NTCP: Normal Tissue Complication Probability, gEUD: generalized Equivalent Uniform Dose.

Given the good results obtained by applying the LKB model to lung DVHs, we went a step further constructing a combined LKB model based on heart and lung irradiation. The combination parameter *α* equal to 0.2 reflects a predominant weight of the lung (figure 6) in this analysis, thus confirming the relevance of lung irradiation in the development of RVD. Predictive power, however, was only mildly increased.

The Spearman’s correlation coefficients and the ROC analysis gave similar values and thus similar prediction performances for all NTCP models (Table 1), with a higher r_s_ and only a slightly higher AUC value for the combined LKB model. However, according to figure 5, the combined LKB model is superior as it assigns patients to high or low risk more effectively than all other NTCP models (LKB-heart, LKB-lung, RS-heart, RS-lung). The data-driven logistic regression model (figure 5f) obtained a similar superior behavior. Also, the previously determined logistic regression model applied to the present dataset resulted in a higher prediction performance (AUC = 0.82, r_s_ = 0.5) compared with all biological NTCP models (AUC values ranging from 0.66 to 0.70).

All together, these results confirm that the heart dose alone cannot be the only critical factor for radiation valvular defects induction. Lung dose may instead contribute significantly, although the mechanism is still to be clarified. In addition, as suggested by the multivariate logistic regression model, the differences in radiation sensitivity between the patients should be also taken into account. Therefore, models based only on critical organ dose may fail to be predictive. In other words, this recalls the concept of biological noise [39] for which all the models are a simplification of more complex biological aspects peculiar to each individual. In the analyzed case, the logistic regression model suggests that the differences in lung and heart volume size may be the key to understand the different individual sensitivity for the development of valvular disease. As already reported in the literature for different radiation-induced toxicity endpoints [40]–[42], a data-driven and exploratory approach to NTCP modeling emerges as a promising and valuable tool to investigate the radiation induced effects in the cardio-pulmonary system given its multivariate intrinsic nature.

In conclusion, we investigated the application of two traditionally accepted NTCP models, namely Lyman-Kutcher-Burman and Relative Seriality, to clinical data for asymptomatic heart toxicity. Parameter estimates were obtained for RVD data fitted separately as a function of heart dose or lung dose. The performance of each prediction model was assessed. A combined heart and lung LKB model was also proposed, resulting in an increased predictive power. Overall, however, a data-driven regression logistic NTCP model outperformed these simpler NTCP models, validating it as a potentially useful and reliable clinical tool for treatment decision making. It is apparently important to have heart and lung volume parameters as part of the prediction model, although the underlying patho-physiological reasons are not well understood and additional studies will be necessary to further clarify them.

## Supporting Information

Table S1 **Summary of clinical and dosimetric variable for heart and lungs, and univariate analysis with incidence of radiation-induced valvular defects.**
(DOC)
