# Supplementary material for: Complication Probability Models for Radiation-Induced Heart Valvular Dysfunction: Do Heart-Lung Interactions Play a Role?
Source: PLoS One. 2014 Oct 31;9(10):e111753. doi: 10.1371/journal.pone.0111753 (PMC4216137; doi:10.1371/journal.pone.0111753)
Supplement: Table S1 — Summary of clinical and dosimetric variable for heart and lungs, and univariate analysis with incidence of radiation-induced valvular defects. (DOC) [file pone.0111753.s001.doc]

|  |  |  |  | Univariate analysis | |
| --- | --- | --- | --- | --- | --- |
|  | Median | Range | | r_s_ | *p-*value |
| **Heart** | | | | | |
| V5 (%) | 79.1 | 0-100 | | .118 | .267 |
| V10 (%) | 75.0 | 0-100 | | .126 | .238 |
| V15 (%) | 72.2 | 0-100 | | .129 | .226 |
| V20 (%) | 70.4 | 0-100 | | .137 | .196 |
| **V25 (%)** | **41.3** | **0-97.1** | | **.222** | **.035** |
| **V30 (%)** | **5.4** | **0-94.4** | | **.260** | **.013** |
| **D_max_ (Gy)** | **31.4** | **0.1-40.9** | | **.312** | **.003** |
| **D_mean_ (Gy)** | **21.0** | **0.1-32.9** | | **.234** | **.027** |
| **Volume (cc)** | **539.3** | **336.0-898.4** | | **.228** | **.030** |
| **Lungs** | | | | | |
| V5 (%) | 40.3 | 3.9-70.1 | | .164 | .123 |
| V10 (%) | 35.7 | 0.6-64.6 | | .148 | .164 |
| V15 (%) | 32.3 | 0-60.9 | | .153 | .151 |
| V20 (%) | 29.6 | 0-57.7 | | .165 | .121 |
| V25 (%) | 24.3 | 0-43.7 | | .208 | .050 |
| **V30 (%)** | **15.7** | **0-38.3** | | **.235** | **.026** |
| **D_max_ (Gy)** | **32.7** | **0-42.1** | | **.333** | **.001** |
| D_mean_ (Gy) | 10.7 | 1.1-17.5 | | .205 | .053 |
| **Volume (cc)** | **2731.1** | **1396.1-5729.4** | | **-.243** | **.021** |

**Table S1**. Summary of clinical and dosimetric variable for heart and lungs, and univariate analysis with incidence of radiation-induced valvular defects

*Abbreviations*: Vx= percentage volume receiving x Gy, Dmax = maximum dose, Dmean = mean dose, r_s_= Spearman’s correlation coefficient

In bold the significant variables.
